# The CtrA phosphorelay integrates differentiation and communication in the marine alphaproteobacterium *Dinoroseobacter shibae*

**DOI:** 10.1186/1471-2164-15-130

**Published:** 2014-02-13

**Authors:** Hui Wang, Lisa Ziesche, Oliver Frank, Victoria Michael, Madeleine Martin, Jörn Petersen, Stefan Schulz, Irene Wagner-Döbler, Jürgen Tomasch

**Affiliations:** Group of Microbial Communication, Helmholtz-Centre for Infection Research (HZI), Braunschweig, Germany; Institute of Organic Chemistry, Technical University Braunschweig, Braunschweig, Germany; Department of Microbial Ecology and Diversity Research, Leibniz Institute DSMZ-German Collection of Microorganisms and Cell Cultures, Braunschweig, Germany

## Abstract

**Background:**

*Dinoroseobacter shibae,* a member of the *Roseobacter* clade abundant in marine environments, maintains morphological heterogeneity throughout growth, with small cells dividing by binary fission and large cells dividing by budding from one or both cell poles. This morphological heterogeneity is lost if the quorum sensing (QS) system is silenced, concurrent with a decreased expression of the CtrA phosphorelay, a regulatory system conserved in *Alphaproteobacteria* and the master regulator of the *Caulobacter crescentus* cell cycle. It consists of the sensor histidine kinase CckA, the phosphotransferase ChpT and the transcriptional regulator CtrA. Here we tested if the QS induced differentiation of *D. shibae* is mediated by the CtrA phosphorelay.

**Results:**

Mutants for *ctrA*, *chpT* and *cckA* showed almost homogeneous cell morphology and divided by binary fission. For *ctrA* and *chpT*, expression in *trans* on a plasmid caused the fraction of cells containing more than two chromosome equivalents to increase above wild-type level, indicating that gene copy number directly controls chromosome number. Transcriptome analysis revealed that CtrA is a master regulator for flagellar biosynthesis and has a great influence on the transition to stationary phase. Interestingly, the expression of the autoinducer synthase genes *luxI*_*2*_ and *luxI*_*3*_ was strongly reduced in all three mutants, resulting in loss of biosynthesis of acylated homoserine-lactones with C14 side-chain, but could be restored by expressing these genes in *trans*. Several phylogenetic clusters of *Alphaproteobacteria* revealed a CtrA binding site in the promoters of QS genes, including *Roseobacters* and *Rhizobia*.

**Conclusions:**

The CtrA phosphorelay induces differentiation of a marine *Roseobacter* strain that is strikingly different from that of *C. crescentus*. Instead of a tightly regulated cell cycle and a switch between two morphotypes, the morphology and cell division of *Dinoroseobacter shibae* are highly heterogeneous. We discovered for the first time that the CtrA phosphorelay controls the biosynthesis of signaling molecules. Thus cell-cell communication and differentiation are interlinked in this organism. This may be a common strategy, since we found a similar genetic set-up in other species in the ecologically relevant group of *Alphaproteobacteria. D. shibae* will be a valuable model organism to study bacterial differentiation into pleomorphic cells.

**Electronic supplementary material:**

The online version of this article (doi:10.1186/1471-2164-15-130) contains supplementary material, which is available to authorized users.

## Background

Bacteria have in the past been regarded as very simple organisms, dividing by binary fission into clones of identical daughter cells which can perform an unlimited number of cell divisions and thus were considered immortal in the absence of external killing events. This view has since been abandoned and replaced by the concept of asymmetric cell division in most if not all bacterial species, resulting in progeny of different cellular composition, different history, and different fate [1]. Asymmetric cell division has first been recognized in those bacterial species that undergo complex differentiation processes, e.g. formation of filaments, buds, spores, swarmer cells and stalked cells. However, even binary fission is asymmetric in many bacteria, including *E. coli* which produces morphologically identical daughter cells. Each daughter cell contains an old and a young cell pole, and this orientation is maintained in subsequent divisions, resulting in an increasing probability of death with increasing number of cell divisions [2, 3]. Like in eukaryotic cells, the number of cell divisions that an individual cell is able to perform appears to be limited in prokaryotic cells, too [1]. Asymmetric cell division through growth from one cell pole has frequently been observed in *Alphaproteobacteria* and recognized as a possibility to create progeny with dissimilar age and cell fates [4].

The freshwater bacterium *Caulobacter crescentus* became the model organism to study asymmetric cell division due to its strictly dimorphic lifestyle. A sessile stalked cell gives birth to a flagellated motile cell that cannot divide until it loses its flagellum and develops a stalk again. The complex molecular mechanisms controlling the cell cycle of *C. crescentus* have been thoroughly analysed [5, 6]. In brief, asymmetry of daughter cells is ensured through a phosphorylation gradient of the essential response regulator CtrA [7] between the two cell poles [8]. This gradient is maintained by the trans-membrane histidine kinase CckA that is localized at the poles in the dividing cell [9]. Different enzymatic complexes maintain its activities as a kinase at the swarmer pole and as a phosphatase at the stalked pole that are transmitted to CtrA via the diffusible phosphotransferase ChpT [10]. This phosphorelay system is conserved in most *Alphaproteobacteria* and has been found to control divergent traits in addition to cell division. The underlying functional principles however are only poorly understood outside of the specialized *Caulobacter* model system [11].

Polar growth is conserved in the order *Rhizobiales*[12] as are most of the cell-cycle regulating genes of *C. crescentus*[11]. CtrA has been demonstrated to be essential in the plant symbiont *Sinorhizobium meliloti*[13] and might play a role during differentiation from free-living, dividing cells into non-dividing, nitrogen fixing and endosymbiotic bacteroids [14]. There is strong evidence that CtrA is also essential for replication and cell division in the plant pathogen *Agrobacterium tumefaciens*[15, 16] as well as in *Brucella abortus*, the causative agent for brucellosis in mammals including humans [17].

In contrast to the aforementioned examples, CtrA has not been found to be essential in the *Rhodospirillales* and *Rhodobacterales* strains examined so far. In both orders only a subset of *C. crescentus* cell-cycle genes is present [11]. The differentiation of the phototroph *Rhodospirillum centeum* from flagellated swarmer cells into non-motile aggregating cysts is controlled by a *ctrA* homologue [18]. Based on evidence from the literature and their finding that CtrA controls flagellar biosynthesis but not cell division in *Magnetospirillum magneticum*, Greene *et al*. suggested that regulation of motility is the primordial role of the CtrA phosphorelay and other functions like control of the cell cycle might have been acquired later during evolution [19]. Indeed, flagella and chemotaxis gene expression but not growth and cell division are impaired in a *ctrA* knock-out mutant of *Rhodobacter capsulatus*[20, 21]. In this organism however, the CtrA phosphorelay also controls expression of a gene cluster coding for the gene transfer agent (GTA) mediating a virus-like exchange of DNA between organisms [21]. As only a small fraction of the population expresses the GTA gene cluster, regulation by CtrA might be heterogeneous or bistable in this case [22]. Another exciting new finding was that expression of *ctrA* depends on the quorum sensing (QS) system of *R. capsulatus*[23]. QS refers to a form of cell-to-cell communication that involves the production, excretion and detection of small diffusible signaling molecules called autoinducers (AI). Thus, the CtrA phosphorelay is integrated into the communication system of this organism.

*Dinoroseobacter shibae* is a representative of the *Roseobacter* clade, an ecologically important phylogenetic cluster of marine *Rhodobacterales*[24]. It was isolated from the dinoflagellate *Prorocentrum lima*[25] and lives in symbiosis with marine algae [26]. *D. shibae* relies on acylated homoserine-lactones (AHL) for communication like many *Proteobacteria*[24]. Genome analysis revealed the presence of three LuxI type AHL synthase genes (termed *luxI*_*1-3*_). *luxI*_*1*_ and *luxI*_*2*_ are located on the chromosome downstream of a gene encoding a LuxR-type transcriptional regulator, whereas *luxI*_*3*_ is on the 86-kb plasmid downstream of a gene encoding an autoinducer but no DNA binding domain [26]. Homologs to the genes *ctrA*, *chpT* and *cckA* have been identified, too [27].

Recently we found that QS induces differentiation of *D. shibae* into pleomorphic cells utilizing distinct types of cell division [27]. A *luxI*_*1*_ knock-out led to a complete loss of AHL biosynthesis, homogeneous cell morphology and faster rate of cell division. Expression of the genes *ctrA*, *chpT* and *cckA* was strongly reduced in this strain. Here we test the hypothesis that the QS induced differentiation of *D. shibae* is mediated by the CckA-ChpT-CtrA phosphorelay system.

## Results

Nine homologs of *C. crescentus* cell cycle control genes can be found in the genome of *D. shibae* (Table 1). Like in most other *Rhodobacterales*, the replication initiator *dnaA*, the proteases *clpP/X*, the DNA-methylase *ccrM*, the transcription factor *gcrA* and the *divL*-*cckA*-*chpT*-*ctrA* phosphorelay are present. Only the latter four genes showed reduced expression in the QS defect mutant [27]. As the putative *divL*-homolog was only weakly conserved and other putative homologs could be found in the genome of *D. shibae*, we concentrated on the analysis of the genes *ctrA*, *chpT* and *cckA*. We constructed single knock-out mutants by replacing these three genes with a gentamycin resistance cassette and compared these strains to the wild-type and the QS deficient strain.

**Table 1:** **Homologs of cell cycle related genes from** ***Caulobacter crescentus*** **NA1000 in** ***D. shibae*** **DFL-12**

Locus Tag ***D. shibae***	Gene Symbol	Description	Locus Tag ***C.crescentus***	Query coverage	E value	Max. identity
Dshi_1644	*cckA*	Integral membrane sensor hybrid histidine kinase	CCNA_01132	50%	2E-103	49%
Dshi_1470	*chpT*	Histidine phosphotransferase	CCNA_03584	89%	2E-16	29%
Dshi_1508	*ctrA*	Two component transcriptional regulator	CCNA_03130	94%	1E-126	74%
Dshi_3433	*divL*	Hypothetical protein	CCNA_03598	46%	3E-25	34%
Dshi_1387	*clpX*	ATP-dependent Clp protease, ATP-binding subunit	CCNA_02039	97%	0E + 00	81%
Dshi_1388	*clpP*	Endopeptidase	CCNA_02041	96%	1E-104	69%
Dshi_2616	*gcrA*	Cell cycle regulator	CCNA_02328	98%	1E-32	40%
Dshi_0024	*ccrM*	DNA methylase N-4/N-6 domain protein	CCNA_00382	94%	2E-179	68%
Dshi_3373	*dnaA*	Chromosomal replication initiator protein	CCNA_00008	98%	1E-115	42%

### Single gene knockouts of *ctrA*, *cckA* and *chpT* reduce the morphological heterogeneity of *D. shibae*

**Figure 1:**
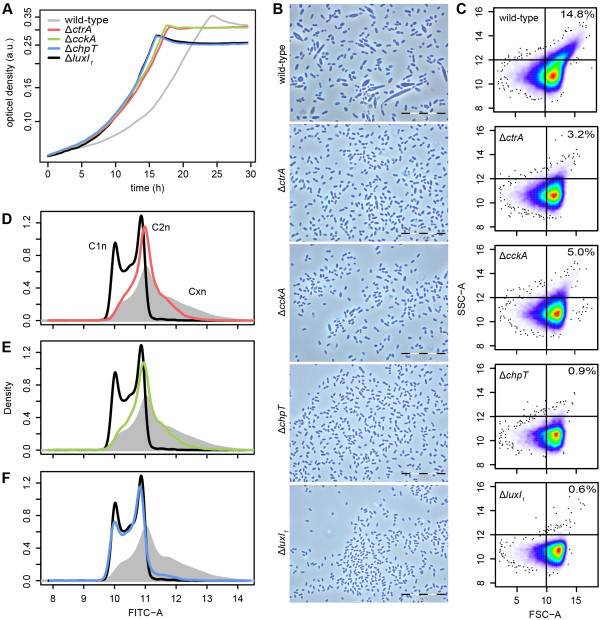
**Phenotypic characterization. (A)** Growth curves of *D. shibae* DFL12 wild-type and mutant strains cultured in minimal see water medium supplemented with 5 mM succinate at 30°C. The y-axis is drawn in ln scale. **(B)** Phase contrast microscopy of corresponding strains. Scale bar represents 20 μm. **(C)** Flow cytometric representation of morphological differences between mutant and wild-type strains based on size (FSC, forward scatter) and granularity (SSC, side scatter). Numbers in plots (top right quadrant) indicate percent larger cells in this area. **(D)** The chromosome equivalent profiles of the *∆ctrA* (red line), **(E)** *∆cckA* (green line) and **(F)** *∆chpT* mutant (blue line) from cultures used in **(A)** were compared to wild-type (grey area) and *∆luxI*_*1*_ (black line) cultures by flow cytometry of SYBR Green I-stained cells (50,000 events counted). The *x* and *y* axes are in log_2_ scale and represent fluorescence intensity and cell density, respectively. The analysis presented in B-F was carried out with cultures in the mid-exponential phase.

All deletion strains were viable and as predicted by our hypothesis their phenotypes were comparable to those of the QS defective mutant. All strains showed a reduced lag phase and increased growth rate in minimal sea water medium supplemented with 5 mM succinate (Figure 1A). Interestingly, the doubling time of the *∆chpT* mutant was almost identical to that of *∆luxI*_*1*_. For *∆ctrA* and *∆cckA* it was slightly longer but still reached only 63% and 65% of the wild type doubling time, respectively (Table 2). Microscopic investigation confirmed the absence of strongly enlarged and elongated cells in all three mutants during exponential growth (Figure 1B). Like in *∆luxI*_*1*_ cells were very homogeneous in size, although a few slightly longer cells were found in the *∆ctrA* and *∆cckA* strains. Scatterplots of flow cytometric analysis of cultures in the mid-exponential phase revealed a reduction of cells with a large forward and sideward scatter – both parameters increase with cell size [28] – in all three mutants (Figure 1C). The number of cells in the upper right quadrant of the plot, representing highly enlarged cells, reached 6%, 23% and 34% of the wild-type for *∆chpT*, *∆ctrA* and *∆cckA*, respectively. These data provide evidence that the CtrA phosphorelay is indeed involved in the control of morphological heterogeneity of *D. shibae*.

**Table 2:** **Growth rate and doubling time of** ***D. shibae*** **wild-type and mutant strains**

Strain	Growth rate ± s.d. (h^-1^)	Doubling time ± s.d. (h)
wild-type	0.16 ± 0.00	4.33 ± 0.07
*∆luxI1*	0.27 ± 0.02	2.61 ± 0.14
*∆ctrA*	0.25 ± 0.01	2.72 ± 0.08
*∆cckA*	0.25 ± 0.01	2.80 ± 0.09
*∆chpT*	0.27 ± 0.01	2.57 ± 0.09

Values represent means and standard deviations of ten biological replicates.

### Replicative diversification is controlled by *ctrA*, *cckA* and *chpT*

**Figure 2:**
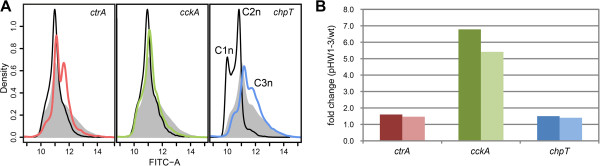
**Flow cytometric analysis of genetically complemented strains. (A)** The chromosome equivalent profiles of the complementation strains ∆*ctrA* pHW1 (red line), ∆*cckA* pHW2 (green line) and ∆*chpT* pHW3 (blue line) are compared to those of the respective knock-out mutants (black line) and the wild-type (grey area). The analysis was carried out with cultures in the mid-exponential phase. **(B)** Differential expression of *ctrA* (red), *cckA* (green) and *chpT* (blue) in the complemented strains (pHW1-3) in *trans* compared to the wild-type strain was tested by RT-qPCR. The results of two biological replicates are shown.

Next we asked whether these mutants had an altered chromosome content as it was revealed for the *∆luxI*_*1*_ strain [27]. We determined the number of chromosome equivalents per cell by stoichiometric DNA staining and subsequent flow cytometric analysis. The strains *∆ctrA* (Figure 1D) and *∆cckA* (Figure 1E) displayed a similar distribution of chromosome equivalents per cell. In both cases the fraction of cells with more than two chromosome equivalents was reduced although not completely absent as in the *∆luxI*_*1*_ strain. In contrast to this finding, the distribution of chromosome equivalents per cell of the *∆chpT* strain was almost identical to that of the QS deficient mutant (Figure 1F). Interestingly, the distribution of the wild-type could not be restored by complementation of any of the mutant strains with the respective gene in *trans* (Figure 2A). Expression of *ctrA* on a low copy plasmid resulted in a larger fraction of cells with three chromosome equivalents than in the wild-type. In the case of *cckA* no changes in the chromosome content distribution compared to the mutant were observed. Expression of *chpT* increased the number of cells with three chromosome equivalents compared to the wild-type. Cells with even higher chromosome content were present, too. Thus, the CtrA phosphorelay is involved in the control of replication in *D. shibae*. In particular it seems to induce replication without cell division, resulting in cells with increased number of chromosomes. Next we compared the expression level of all three genes in the complemented strains using RT-qPCR. Whereas *ctrA* and *chpT* showed an expression level only 1.5 fold higher than in the wild-type, cckA was six fold over-expressed (Figure 2B). Thus, the differences in chromosome content between wild-type and complemented mutant could be explained by the slightly higher expression level of *ctrA* and *chpT* in *trans*. For the failure of overexpressed *cckA* to influence the chromosome content of the mutant strain other explanations have to be found.

### Comparative transcriptome analysis revealed substantial overlaps of gene expression in *ctrA*, *cckA* and *chpT*-deficient strains

To understand the global contribution of the CtrA phosphorelay to gene regulation in *D. shibae*, we performed microarray analysis to examine gene expression profiles of the three mutant strains compared to the wild-type in both mid-exponential and stationary phase of growth. Differential expression was assumed for genes with an absolute log_2_ fold change higher or equal to one and a Benjamini-Hochberg-adjusted p-value smaller than 0.05. The log_2_ fold-changes and p-values for all genes present on the microarray can be found in Additional file 1: Table S1, also including the microarray data for *∆luxI*_*1*_ versus wild-type [27] for comparison. The differential expression of four genes was also confirmed by quantitative reverse transcription PCR (qRT-PCR). This data can be found in supplementary Additional file 2: Figure S1.

**Figure 3:**
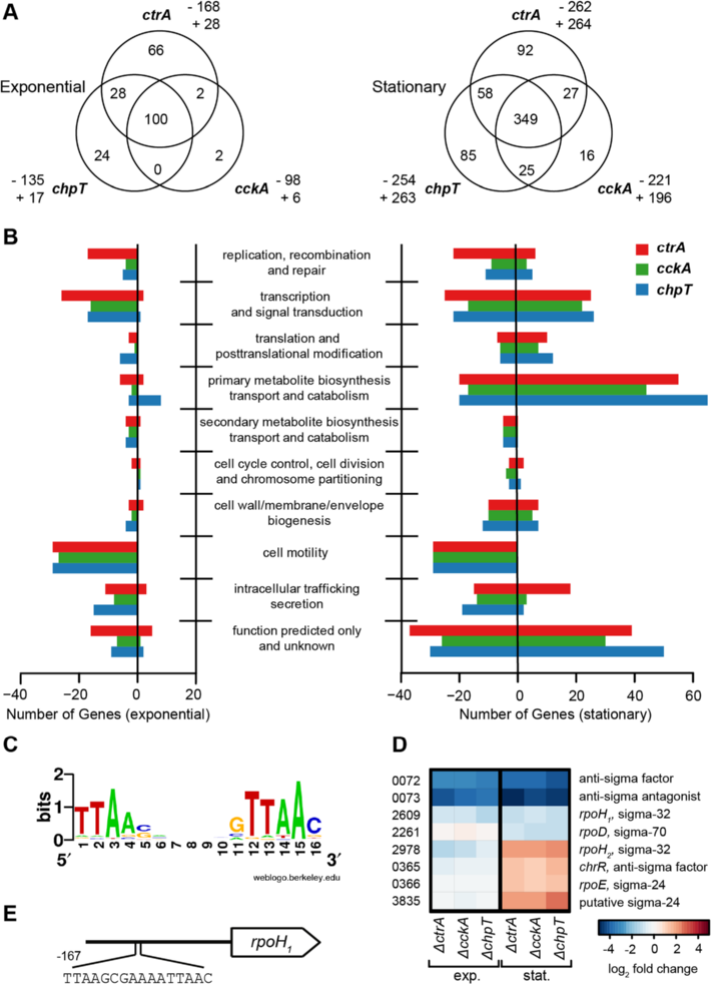
**Overview of microarray analysis in the three mutants in the logarithmic (left) and stationary (right) phase of growth. (A)** Venn diagrams showing numbers of genes differentially expressed in the indicated mutants and having at least a two-fold change with cut-off *p* ≤ 0.05 compared to the wild-type both in exponential and stationary phase. Numbers represent genes changed exclusively in one mutant, while the numbers in the intersections represent those occurring between each two or among all the three mutants. Pairwise numbers show genes regulated in one mutant. “+”: up-regulation; “-”: down-regulation. **(B)** Functional categories of regulated genes based on clusters of orthologous groups (COGs) both in exponential and stationary phase. Functional categories were determined using COG for *D. shibae* provided by IMG (http://img.jgi.doe.gov/). The bars represent the number of regulated genes within a given category. *ctrA*: red; *cckA*: green; *chpT*: blue. (Genes that were not assigned in COG are not included here but shown in Additional file 1: Table S1). **(C)** Sequence logo of the putative CtrA binding site in the *D. shibae* genome. This motif logo was plotted using Weblogo (weblogo.berkeley.edu). **(D)** Heat map showing gene expression of (anti-)sigma factors. The color bar represents the expression level in log_2_ scale. Red indicates relatively high expression levels; blue indicates relatively low expression levels. **(E)** A putative CtrA binding site located in the promoter region of *rpoH*_*1*_.

During the exponential growth phase 196 genes (28 up, 168 down) were differentially expressed in the *∆ctrA* mutant in comparison to the wild-type strain. In the *∆cckA* mutant 104 genes (6 up, 98 down), and in *∆chpT* mutant 152 genes (17 up, 135 down) were differentially expressed (Figure 3A). A total of 100 genes were differentially expressed in all three mutant strains, among which only 5% with increased, but 95% with decreased expression. Thus, the CtrA phosphorelay acts as an activator of gene expression during exponential growth.

In the stationary phase, the number of genes that were differentially expressed increased remarkably (Figure 3A). 526, 417 and 517 genes were differentially expressed in the *∆ctrA*, *∆cckA* and *∆chpT* mutant, respectively. In all three cases around half of the genes were up-regulated compared to the wild-type. 349 genes were differentially expressed in all three mutants. The large overlap in gene expression of all three mutant strains suggests that CckA, ChpT and CtrA indeed act in a common pathway in *D. shibae*.

Differentially regulated genes were categorized by Clusters of Orthologous Groups (COG) designations, and the numbers of genes differentially regulated both in exponential and stationary phase in each category are shown in Figure 3B. Categories that showed a notable over-representation of down-regulated genes in both growth phases included those related to cell motility, intracellular trafficking and secretion, and transcription and signal transduction. Specific groups of the CtrA regulon will be discussed in later sections. The CtrA binding site was well conserved in *D. shibae* and could be found in 101 promoter regions (Figure 3C and Additional file 3: Table S2).

### The *ctrA* phosphorelay influences the adaptation of *D. shibae* to stationary phase

The enormous increase in the number of differentially expressed genes in the stationary phase was mainly due to the higher expression of genes falling in the categories primary metabolite biosynthesis, transport and catabolism as well as genes with a function in transcriptional control and signal transduction (Figure 3B). In particular many genes involved in amino acid biosynthesis and transport showed a stronger expression in all three mutants. Remarkably genes encoding the cytochrome c complex, a cytochrome bd ubiquinol oxidase as well as two other cytochromes were higher expressed indicating that the redox state of mutant cells may be altered and they may adapt to stationary phase in a different way. The activation of various genes predicted to encode heat- and cold-shock proteins as well as chaperones indicates a stress response in the mutant strains (Additional file 1: Table S1). We asked next which regulatory genes might be involved in the different adaptation strategies. Two sigma factors (*rpoH*_*2*_ and a putative sigma-24 factor) and one sigma/anti sigma factor pair (*rpoE* and *chrR*) showed up-regulation in all three mutants exclusively in stationary phase (Figure 3D). These *bona fide* regulators of the oxidative stress response [29] therefore represent likely candidates for the observed induction of gene expression in the stationary phase. None of them had a CtrA binding site in their promoter region. The only sigma factor with a respective binding site was *rpoH*_*1*_ (Figure 3E) which showed reduced expression in all three mutants during both growth phases (Figure 3D). Thus, the differences between wild-type and mutants in the stationary phase might not be a result of direct regulation by the CtrA phosphorelay.

Could phenotypic observations help to interpret these data? We found that the wild-type cells were still heterogeneous in the stationary phase, although cell size was on average lower than in the exponential growth phase. In contrast, mutant strain cells – small rods in the exponential phase – were more cocci-shaped in the stationary phase (Additional file 4: Figure S2A). Flow cytometric analysis confirmed the overall size reduction of all strains (Additional file 4: Figure S2B). Determination of the number of chromosome equivalents per cell revealed a shift of the peaks to 20% lower intensity levels (Additional file 4: Figure S2C). As this shift was not stoichiometric we assume that it was caused not by a reduction of chromosome equivalents but rather by a reduction of the RNA content in the cells. RNA has not been removed prior to staining and is also stained by SybrGreen. Furthermore the fraction of cells with only one chromosome equivalent was substantially higher for all strains. It was highest for the *∆luxI*_*1*_ mutant, followed by *∆chpT*, *∆cckA*, *∆ctrA* and the wild-type. The latter was also the only strain for which cells with more than two chromosome equivalents could still be found. Interestingly, some cells of the *∆ctrA* and *∆cckA* mutant strains formed long chains indicating that they could not divide properly (Additional file 4: Figure S2A). Both, transcriptome and phenotypic data suggest that the QS induced and CtrA phosphorelay mediated pleomorphism substantially alters the adaptation of *D. shibae* to nutrient limitation.

### The *ctrA* phosphorelay regulates the expression of a gene transfer agent gene cluster

Gene transfer agents (GTAs) are phage-like particles that have first been discovered in *Rhodobacter capsulatus*[30]. In *D. shibae*, we have found an approximately 15 kb GTA gene cluster (Dshi_2162-2178) with identical organization like in *R. capsulatus*. Our microarray results showed a slightly but consistently reduced expression of the complete GTA gene cluster in all three mutant strains. Two genes of this cluster (Dshi_2174, Dshi_2175) were significantly down-regulated in all mutants (Additional file 1: Table S1). In *R. capsulatus*, CckA and CtrA have been shown to positively regulate the expression of the GTA structural genes [31]. However, only 0.15% of all cells actively expressed this cluster [22]. Assuming a similar frequency in *D. shibae*, it is likely that down-regulation of the GTA cluster might not result in a strong signal for transcriptome analysis of the whole culture.

### CtrA is a master regulator of flagellar biosynthesis

**Figure 4:**
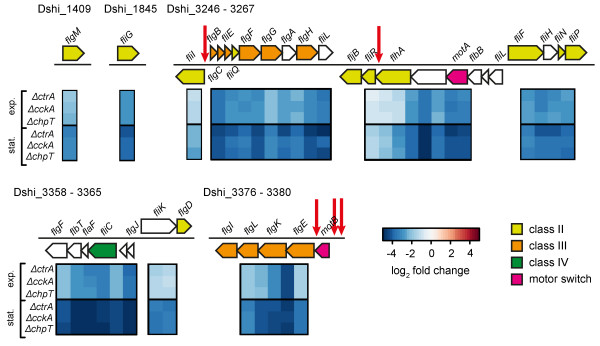
**Regulation of flagellar biosynthesis.** Heat map showing the expression of flagellar genes with corresponding genomic context. Color bar as in Figure 3. Arrows indicate ORFs and the direction of transcription. Gene names are indicated above or below the respective ORFs. ORFs without gene names represent genes annotated as hypothetical protein. Colored arrows represent flagellar genes belonging to class II (yellow), class III (orange), class IV (green) and motor switch complex (pink). Open arrows represent ORFs with unknown classification. Vertical red arrows indicate the position of putative CtrA binding sites found in the promoters of some flagella genes.

The most striking group of differentially regulated genes that were significantly changed in both growth phases were genes associated with cell motility (Figure 3B). In the *D. shibae* genome 37 predicted flagellar genes are present. The majority of them are located in three gene clusters (Dshi_3246-3267, Dshi_3358-3365, Dshi_3376-3380). Two additional genes (Dshi_1409 and Dshi_1845) are located at two separate positions in the chromosome. Microarray analysis showed that all the flagellar genes were down-regulated in the *∆ctrA* mutant compared to the wild-type strain (Figure 4). Flagella genes are categorized into four functional classes which are regulated in a hierarchical way [32]. Class I genes encode the master regulators. In *C. crescentus* the *ctrA* gene belongs to class I which is known to activate class II flagellar genes, whose products are required for the expression of later flagellar genes [33]. To investigate if CtrA might similarly be a class I flagellar regulator in *D. shibae*, we carried out prediction of CrtA binding sites in the *D. shibae* genome using the well described consensus sequence (TTAA-N_7_-TTAAC) from *C. crescentus*[11]. The results revealed that five flagellar genes possess a CtrA binding motif. They are *fliI* (Dshi_3246) and *fliR* (Dshi_3257) of the class II flagellar genes, as well as *flgB* (Dshi_3247) and *flgE* (Dshi_3379) of the class III flagellar genes and one gene encoding for a motor switch protein (Dshi_3380). These findings indicate that like in *C. crescentus ctrA* could belong to the class I flagellar gene and appears to be a master regulator of flagella gene expression in *D. shibae*. Microarray analysis also showed reduced gene expression of all flagellar genes in the *∆cckA* and *∆chpT* mutants compared to the wild-type strain. The histidine kinase CckA and the phosphotransferase ChpT are required for phosphorylation of CtrA. These results suggest that phosphorylated CtrA is essential for the expression of flagellar genes.

### Plasmid gene expression in the *∆ctrA* mutant strain

Genes with a significant change in expression in all three mutants were located on the chromosome, with four exceptions, among them the *luxI*_*3*_ gene. This picture changed when only those genes were taken into consideration that were regulated exclusively in the *∆ctrA* strain. Two of the five plasmids of *D. shibae*, pDshi01 (NC_009955) and pDshi03 (NC_009957), are in large parts syntenic (103 kb shared with 90% identity) and therefore regarded as sister plasmids of common origin [26]. Despite this strong similarity, they differ with respect to their replication operons which are from different compatibility groups [34]. We found 43 genes on pDshi01 being down-regulated only in *D. shibae ∆ctrA*. In particular, the replication and toxin/antidote system showed a strong loss in the microarray signal (Additional file 5: Figure S3A). Plotting the expression changes of all genes against a theoretical normal distribution showed that the log_2_ fold changes were biased towards lower expression of pDshi01 (Additional file 5: Figure S3B). The observed bias in the expression changes of this plasmid leaves room for two different explanations. It could mean that CtrA acts exclusively as an activator of pDshi01 gene expression which is highly unlikely given the high degree of identity between pDshi01 and pDshi03. More likely, this bias could also explained by a modulation of the copy number or loss of this plasmid in this mutant. PCR using pDshi01 specific primers and genomic DNA as a template demonstrated that indeed the plasmid has been lost in the *∆ctrA* knock-out strain (Additional file 5: Figure S3C). Given the strong overlap in gene expression changes between and consistent phenotypic alterations of all three mutants we assumed that plasmid loss has no great impact on the experimental results regarding the *∆ctrA* mutant. However, as plasmid loss can also occur randomly, it should not be ascribed to the loss of CtrA activity.

### Regulation of *C. crescentus* cell-cycle homologs by the CtrA phosphorelay

**Figure 5:**
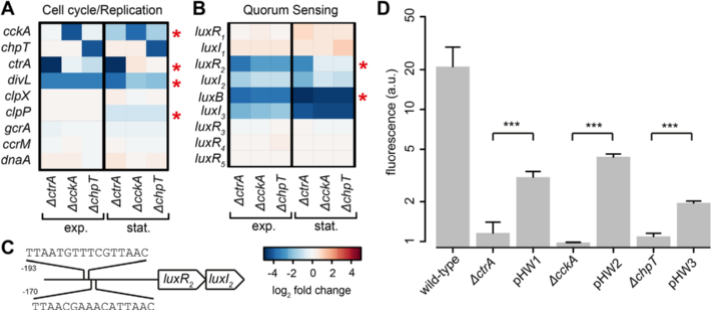
**Regulation of cell cycle related genes and QS genes. (A)** Heat map showing gene expression of cell cycle related genes and **(B)** QS genes. The autoinducer binding domain gene Dshi_4067 has been named *luxB*. Red stars indicate putative CtrA binding sites found at the promoters of these genes. Color bar as in Figure 3. **(C)** The promoter region of the LuxR_2_I_2_ operon showing putative CtrA binding sites. **(D)** Production of long-chain AHLs of *D. shibae* wild-type, knock-out mutant strains and their respective genetic complementations *∆ctrA*pHW1 (short: pHW1), *∆cckA*pHW1 (short: pHW2) and *∆chpT*pHW1 (short: pHW3). Results are presented as fold induction obtained by dividing the specific fluorescence intensity of the samples by that of the negative control (DCM). Values represent means and standard deviations of three biological replicates. Asterisks indicate statistically significant differences between complementation strain and respective knock-out mutant calculated using Student’s *t* test (***p < 0.001).

Transcriptome data for the nine homologs to the *C. crescentus* cell cycle regulators are summarized in Figure 5A. Deletion of *ctrA* did not affect the expression of *cckA* and *chpT* in the exponential growth phase whereas *cckA* expression was reduced in this mutant in the stationary phase. Deletion of *cckA* did not affect the expression of *ctrA* and *chpT* in both growth phases. Deletion of *chpT* caused reduced expression of *ctrA* in the exponential phase and of *cckA* in the stationary phase. Thus, the self-regulation of the CtrA phosphorelay genes seems to be dependent on the growth phase. Of the other homologs of *C. crescentus* cell cycle regulators only the putative *divL* homolog showed a reduced expression in all three mutants. Binding sites for CtrA were found in the promoter of *ctrA* itself, suggesting an auto-regulatory loop for this gene. Binding sites were also identified in the promoters of *cckA*, *divL* and *clpP*. The latter showed no response in any of the knock-outs, indicating that additional mechanisms are involved in the regulation of this gene.

### The CtrA phosphorelay induces autoinducer biosynthesis

Recently we found that knock-out of the autoinducer synthase *luxI*_*1*_ led to a reduced expression of *ctrA*, *cckA*, *chpT, divL* as well as the QS genes *luxR*_*2*_*I*_*2*_ and the AHL synthase *luxI*_*3*_ during exponential growth and stationary phase, resulting in a complete loss of autoinducer biosynthesis [27]. Here, we found that the latter QS genes were also down-regulated in the CtrA phosphorelay mutants in both growth phases (Figure 5B). Expression of *luxR*_*2*_*I*_*2*_ was most strongly reduced in the *∆ctrA* strain and for all three strains expression was lower in the exponential phase. By contrast, the down-regulation of the AHL synthase *luxI*_*3*_ and the autoinducer binding domain gene Dshi_4067 which are located on the 86 kb plasmid pDshi04 (NC_009958) was more pronounced in the stationary phase. The gene expression of *luxI*_*1*_ (Dshi_0312) and its cognate *luxR*_*1*_ regulator (Dshi_0311) was not affected by deletion of any of the three genes in the CtrA regulon. The same was true for the three orphan *luxR*-type regulators (Figure 5B) whose function is currently unknown. We found two perfect CtrA binding sites in the promoter of *luxR*_*2*_ and one conserved binding site in front of Dshi_4067 suggesting that CtrA is tightly integrated into the control of AHL biosynthesis (Figure 5C). Therefore we investigated the AHL production using the biosensor strain *P. putida* F117 pKR-C12 which responds mainly to long-chain AHLs [35]. Surprisingly, the extracts of all three mutant strains failed to induce fluorescence in the reporter strain (Figure 5D). In contrast, we were able to detect the production of AHLs in the genetic complementation strains of all the knock-out mutants, although with a reduced amount compared to the wild-type.

All three autoinducer synthases of *D. shibae* produce long-chain AHLs, as demonstrated by heterologous expression in *E. coli* and *D. shibae* followed by GC-MS analysis [36]. However, LuxI_1_ is the only one producing the C18en-HSL and C18dien-HSL which are the most powerful AI in *D. shibae* and which also trigger expression of *luxI*_*2*_ und *luxI*_*3*_. Since the transcription of *luxI*_*1*_ was unchanged in the mutants, but transcription of *luxI*_*2*_ and *luxI*_*3*_ was reduced, we hypothesize that the AHLs produced by LuxI_2_ or LuxI_3_ contributed most to the response of the sensor strain *P. putida* F117 pKR-C12. The luxR transcriptional regulator of this sensor strain is derived from the *las*-operon of *P. aeruginosa* and is most sensitive for 3-oxo-C12-HSL. GC-MS analysis of the extracts confirms this hypothesis (Table 3 and Additional file 6: Figure S4). C18en- and C18dien-HSL as well as C14en- and 3-oxo-C14-HSL could be detected in extracts derived from a culture of wild-type *D. shibae*. However, only the AHLs with a C18 side-chain could be detected in all three mutant strains. 3-oxo-C14-HSL could only be detected in the complemented *∆cckA* mutant strain. This strain also showed the highest signal in the bioassay except for the wild-type. Therefore we assume that this AHL might also be produced in the complemented *∆ctrA* and *∆chpT* strains but the concentration was below the detection limit of the GC-MS analysis.

**Table 3:** **AHLs in different strains of** ***D. shibae*** **identified by GC-MS**

Strain	C14en-HSL	3-oxo-C14HSL	C18en-HSL	C18dien-HSL
Wild-type	x	x	x	x
∆*ctrA*			x	x
∆*ctrA*pHW1			x	x
∆*cckA*			x	x
∆*cckA*pHW2		x	x	x
∆*chpT*			x	x
∆*chpT*pHW3			x	x

### Integration of the CtrA phosphorelay and quorum sensing might be present in other *Alphaproteobacteria*

**Figure 6:**
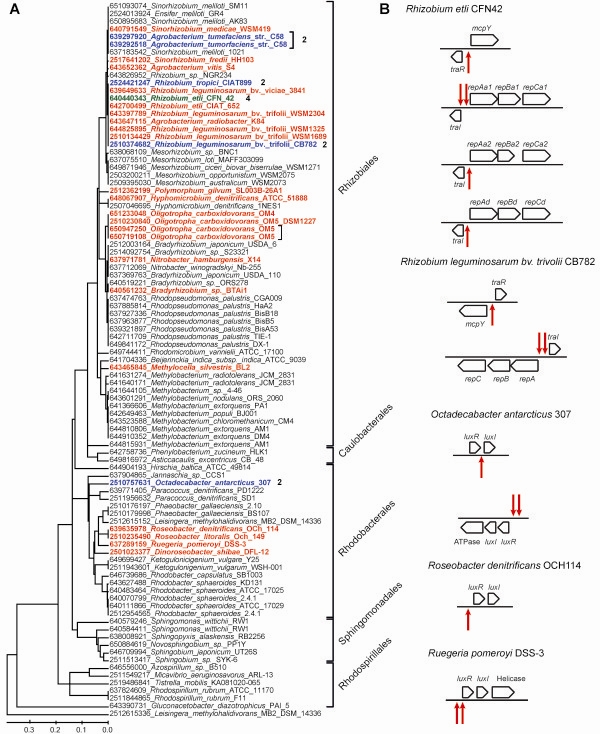
**Evolutionary relationships of response regulator CtrA and QS genes in alpha-proteobacteria. (A)** Neighbor-joining tree of CtrA homologs based on 179 amino acid positions excluding alignment gaps. Here shown are all 89 alphaproteobacterial strains for which complete genome information was available from IMG (http://img.jgi.doe.gov/) and that have both *ctrA* and an autoinducer synthase in their genome. Black, red, blue and green represent strains without, with one, with two and four putative CtrA binding sites in the promoters of either an autoinducer synthase or autoinducer –binding transcription factor gene, respectively. Numbers in front of the name are IMG unique identifiers. **(B)** Genetic organization of *luxI/R* or the homologs *traI/R* with putative CtrA binding sites indicated as red arrows in several selected strains from **(A)**.

Although the control of *ctrA* expression by a LuxR-type transcription factor has recently been demonstrated in *R. capsulatus*[23] no case in which CtrA regulates QS genes has been reported yet. We wondered if this regulatory relationship might also be present in other *Alphaproteobacteria*. We selected 89 finished genomes of *Alphaproteobacteria* that harbor both the *ctrA* gene and at least one gene coding for an autoinducer synthase. We searched for CtrA binding sites in the promoters of all genes that have either an autoinducer synthesis (pfam00765) or an autoinducer binding (pfam03475) domain. We constructed a neighbor joining tree of the identified CtrA proteins in order to test whether there is a relationship between the evolution of CtrA and the evolution of its integration into QS systems. We found that – among the selected genomes – CtrA binding sites in front of QS genes were restricted to the orders *Rhizobiales* and *Rhodobacterales* and also scattered in these orders (Figure 6A). In particular, this constellation was found in several *(Brady-)Rhizobiaceae* strains but absent from the *Methylobacteriaceae* family and the genus *Rhodopseudomonas*. Inside the order *Rhodobacterales*, putative CtrA regulation of QS was restricted to some marine *Roseobacter* strains. Examples of the genome environment are depicted in Figure 6B. One outstanding example is *Rhizobium etli* CFN42 with identified CtrA binding site in front of four QS genes. Three of them are located on plasmids, and the autoinducer synthase gene is located upstream of the repABC-type partitioning system. Another binding site was found in a bidirectional promoter between an autoinducer binding transcription factor and a chemotaxis protein encoding gene. The same constellation was also found for *Rhizobium leguminosarum bv. trifolii* CB782. It has already been shown that QS controls plasmid replication in Rhizobiales [37]. The marine *Roseobacter* species *Octadecabacter antarcticus* 307 has two *luxR/luxI* pairs encoded in its genome. The first one has one binding site in front of the *luxI* gene, the second has two binding sites in front of the *luxR* gene. In contrast *Ruegeria pomeroyi* and *Roseobacter litoralis* have only one *luxR/luxI* pair with two and one CtrA binding site, respectively. The scattered occurrence and the different ways how CtrA is integrated into QS control suggest that this regulatory mechanism has evolved several times independently in *Alphaproteobacteria*.

## Discussion

**Figure 7:**
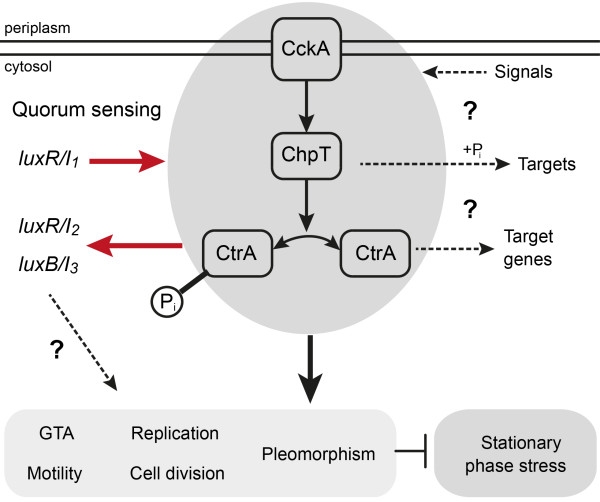
**Model of the CtrA phosphorelay regulated traits in** ***D. shibae*** **.** Based on the data presented in this work and in (Patzelt et al. [27]), we propose that the CtrA-phosphorelay is functional and integrated into the complex QS system of *D. shibae*. The luxR/I_1_ system controls expression of the phosphorelay genes. Phosphorylated CtrA activates expression of the other QS genes and is responsible for several cellular processes in *D. shibae*, i.e. pleomorphic development, cell division, GTA, and motility. Some of these traits might integrate signals from both, CtrA and LuxR/I. In addition, stationary phase stress is higher in cells lacking the phosphorelay activity. The phosphorelay might also integrate other signals as QS. ChpT might also be involved in phosphorylation of other targets in the cell. Unphosphorylated CtrA may also play a role in gene regulation.

17 years of thorough research have brought to us a comprehensive understanding of how the CtrA phosphorelay tightly controls cell cycle progression and differentiation in the strictly dimorphic *Caulobacter crescentus*. Here we studied the role of this regulatory system in a marine bacterium that represents the antithesis to this well-studied model organism. Growth and cell-division of *D. shibae* seem to be totally chaotic – as long as the cells are able to communicate. Cell size and morphology are very heterogeneous in the wild-type and cells divide by binary fission as well as budding from one or both cell poles. Heterogeneity in cell-size and type of cell division is lost in a QS deficient strain [27]. We now have demonstrated that the QS induced pleomorphic lifestyle is controlled by the CtrA phosphorelay. The regulated traits are summarized in Figure 7 and will be discussed in the following sections.

Knock-out of *ctrA*, *cckA* and *chpT* resulted in a similar reduction of morphological heterogeneity and cell size as in the QS mutant confirming that they act in a common pathway controlling cell growth. We further demonstrated that all three genes are involved in the control of replication as their loss led to reduction of cells with a larger number of chromosomes in the population. This was not unexpected as cell size and replication are coordinated in bacteria [38]. In contrast to *ctrA* and *chpT* genetic complementation of *∆cckA* failed to shift the population towards cells with higher chromosome content. As the gene was transcribed and autoinducer synthesis could be restored in *trans*, it is unlikely that the introduced plasmid was not functional at all. The sensor histidine kinase is a trans-membrane protein. In *C. crescentus* it is specifically localized at the cell poles and creates a gradient of phosphorylated CtrA [39]. In *D. shibae*, the six fold over-expression of cckA might lead to a mislocation of the protein and therefore a gradient that might be necessary for differentiation into cells with multiple chromosomes could not be formed.

The phenotype of the *∆chpT* mutant – in contrast to *∆ctrA* and *∆cckA –* is almost identical to that of the *∆luxI*_*1*_ mutant. However, comparison of the transcriptome data from *∆chpT* and *∆luxI*_*1*_[27] reveals only few genes that are differentially expressed in both strains but not in *∆ctrA* and *∆cckA* (Additional file 1: Table S1). Among them are genes encoding parts of an ATP synthase (Dshi_3028-3031) that are up-regulated and T4SS (Dshi_3975/3976) that are down-regulated in both mutant strains as well as few hypothetical genes. However, these data offer no explanation for the physiological differences between the aforementioned strains. In *C. crescentus*, CtrA is not the only target of phosphorylation by ChpT [10] and the differences between *∆chpT* and *∆luxI*_*1*_ (in the latter the whole phosphorelay is down-regulated) on one side and *∆ctrA* and *∆cckA* on the other might be a result of altered phosphorylation states rather than gene expression.

The remarkable variety of cell shapes and sizes as well as chromosome copy numbers of *D. shibae* naturally raises three questions: How is this heterogeneity induced, are there other traits that are heterogeneously regulated as well and finally, how does the population benefit from such heterogeneity? At the moment we can only speculate on the mechanisms underlying the development of heterogeneous cells. It might be stochastic gene expression or a bistable regulatory feedback loop (reviewed in [40] and [41]). Single cell techniques have to be developed to answer this question. We showed that the control of flagellar biosynthesis by CtrA is also conserved in *D. shibae*. Recently it has been demonstrated that under the same growth conditions that have been used for experiments in this manuscript, only a fraction of *D. shibae* cells are motile. Thus flagellar biosynthesis seems to be heterogeneously distributed, too [42]. Furthermore it seems reasonable that the GTA cluster in *D. shibae* is heterogeneously activated like in *R. capsulatus*[32].

Differentiation clearly represents a burden for population growth of *D. shibae*, as the doubling time is longer and the maximum number of cells is reduced [27]. In this context one might ask if it also represents a burden during adaptation to stationary phase. Our observations suggest that *D. shibae* reduces its cell size in response to nutrient depletion like marine Vibrios [43]. This cell-size reduction is of course easier to achieve if the cells are already small on entry to stationary phase, and should reduce the need for costly cellular reprogramming. However, our data also suggest that the fast dividing mutant cells might be subjected to higher oxidative stress than the slower growing wild-type population. Thus, fast growth in the exponential phase could also represent a burden during adaption to stationary phase. The role of the CtrA phosphorelay for adaptation to nutrient depletion clearly needs more in depth analysis.

Our recent paper on the role of the LuxI_1_ autoinducer synthase [27] together with the present work suggest a strong integration of QS and the CtrA phosphorelay. Our hypothesis was that LuxR_1_ and LuxI_1_ are at the top of a hierarchical regulatory system that activates expression of CtrA phosphorelay genes, the *luxR*_*2*_*I*_*2*_ operon and the plasmid encoded *luxI*_*3*_. The present findings suggest that CtrA actually activates *luxR*_*2*_*I*_*2*_ and *luxI*_*3*_ expression; consequently the phosphorelay is not only regulated by but actually integrated into the QS system. Thus, the CtrA mediated differentiation leads to enhanced autoinducer production. This has not been reported for any other organism so far, but our promoter-analysis in *Alphaproteobacteria* predicts that this kind of integration of these two regulatory systems might indeed be widespread. The question how the cell or the whole population benefits from this regulatory constellation will be addressed in future research.

## Conclusion

It is now recognized that QS is not only employed to coordinate the behavior of a bacterial population but often induces heterogeneous responses towards signaling molecules. This has for example also been demonstrated for the marine pathogen *Vibrio harveyi*[44, 45] and the marine symbiont *Vibrio fischeri*[46]. *D. shibae* is an outstanding organism as its heterogeneity is manifested as a pronounced morphological pleomorphism. The fact that loss of morphological heterogeneity results in faster growth will be beneficial for screening libraries of mutants in order to identify genes that are involved in this differentiation process. Studying this organism in more detail will also enhance our understanding of the evolution of CtrA phosphorelay mediated control of cellular polarity, which in the case of *C. crescentus* leads to a tightly controlled cell cycle and in the case of *D. shibae* leads to high heterogeneity of replication and cell division.

## Methods

### Bacterial strains and growth conditions

The strains and plasmids used for this study are listed in Additional file 7: Table S3. *D. shibae* DFL-12 strains were grown at 30°C and 160 rpm in half-concentrated Marine Broth or on the same medium solidified with 1.5% agar. Alternatively, cells were grown in a chemically defined sea water medium (SWM) supplemented with 5 mM succinate, prepared as described previously [29]. When appropriate, 150 μg/ml of gentamicin or 500 μg/ml of kanamycin was added. *Escherichia coli* strains were grown at 37°C and 180 rpm in Luria-Bertani (LB) broth or on LB agar supplemented with ampicillin (100 μg/ml), gentamicin (20 μg/ml), kanamycin (50 μg/ml), and/or aminolevulinic acid (50 μg/ml) if necessary.

### Construction of *D. shibae* deletion mutants

For deletion of *ctrA* (Dshi_1508), *cckA* (Dshi_1644), and *chpT* (Dshi_1470), each full-length ORF with flanking regions (approximately 800 bp on either side) was amplified by PCR using *D. shibae* DFL12 genomic DNA and appropriate primers (Additional file 8: Table S4). These fragments were cloned into the multiple cloning site of pEX18Ap, a suicide plasmid carrying an ampicillin resistance cassette. From the resulting plasmids, the target ORFs were replaced with a gentamicin resistance cassette from pBBR1MCS-5 [47] using the CloneEZ PCR Cloning Kit (GenScript, Piscataway, USA) according to the manufacturer’s instructions. The resulting constructs were transferred to *D. shibae* DFL12 using conjugation with *E. coli* ST18 as a helper strain [48] and integrated into the wild-type strain by double-homologous recombination. The transconjugants were selected on half-concentrated Marine agar plates containing gentamicin. All mutant strains constructed in this study were confirmed by PCR and sequencing.

### Complementation of mutants

Complementation of the deleted genes under the control of their native upstream sequences was performed with the broad-host-range vector pBBR1MCS-2 [47]. The complementing fragments were amplified by PCR using appropriate primers (Table 2) and cloned into pBBR1MCS-2, generating plasmids pHW1, pHW2 and pHW3. These plasmids were transferred into the respective mutant strain via conjugation using *E. coli* ST18 [49]. All complementations were also confirmed by PCR and sequencing.

### Microscopy

Cell morphology was observed with an Olympus BX60 microscope with a Plan 100×/1.25 oil immersion objective. All images were acquired using the Olympus OM-4 Ti camera and the cell B image acquisition software (Olympus, Japan).

### Growth curves

For growth curves, cell material from half-concentrated MB plates was inoculated into 20 ml of SWM + 5 mM succinate and incubated overnight at 30°C and 160 rpm to obtain a pre-culture. Optical density at 600 nm (OD_600_) was measured with spectrophotometer Ultrospec 3100 pro (Biochrom Ltd, Cambridge CB4 0FJ England). Cultures were diluted to 0.01 OD_600_ and then 200 μl were placed into each well of a Honeycomb 2 plate (100 wells each plate, Oy Growth Curves Ab Ltd, Helsinki, Finland). OD_600_ was monitored every 30 min for 36 hours in the automated microbiology growth analysis system Bioscreen C (Oy Growth Curves Ab Ltd, Helsinki, Finland). Growth curves were plotted in ln scale using the R program.

### Sampling

Cultures for the flow cytometry and microarray analysis were inoculated into 100 ml medium in 300 ml Erlenmeyer flasks with an initial OD_600_ of 0.01 from overnight cultures and incubated for 36 hours. Samples of the wild-type and all mutant strains were taken in the middle of the exponential phase at an OD_600_ of approximately 0.4 and in the stationary phase 6 h after OD_600_ had reached a maximum.

### Flow cytometry

Samples for flow cytometry analyses were fixed at a final concentration of 2% glutaraldehyde and stained with SYBR Green I (Molecular Probes, Leiden, The Netherlands) by diluting the stock reagent 1: 10,000 into the samples. For each experiment, the DNA content was measured in a population of 50, 000 cells using the BD FACS Canto analyzer. The data were analysed using the ‘flowCore’ package [50] of the R BioConductor project.

### RNA isolation

Cells were collected by centrifugation at 12,000 × g for 1 min at 4°C, covered with 1 ml Trizol reagent (Ambion, Germany), immediately frozen in liquid N_2_ and stored at -70°C until processing. For RNA extraction, cells were homogenized with ~ 0.3 g of glass beads in the FastPrep-24 instrument (MP Biomedicals, California, USA) at 6.0 m/s for 3 min and then incubated for 5 min at room temperature. Samples were centrifuged at 12,000 × g for 10 min at 4°C and the supernatants were transferred to fresh tubes, followed by the addition of 100 μl of 1-bromo-3-chloropropane (BCP, Sigma, Germany) and incubation for 10 min at room temperature. Samples were centrifuged at 12,000 × g for 10 min at 4°C, after which the aqueous phase was transferred to new tubes and mixed with 500 μl of absolute ethanol. Extracts were applied to RNeasy spin columns (RNeasy mini kit, Qiagen, Hilden, Germany) and processed according to the manufacturer’s instructions. In addition, samples were treated with DNAse I (Qiagen, Hilden, Germany). Removal of genomic DNA was verified via PCR. The concentration of the RNA was quantified using a NanoDrop spectrophotometer (Peqlab, Erlangen, Germany) and the RNA integrity was assessed using Bioanalyzer 2100 (Agilent, Santa clara, USA).

### Microarray experiment

2 μg of total RNA was labeled with Cy3 or Cy5 using the ULS-system (Kreatech, Amsterdam, The Netherlands) according to the manufacturer's protocol. 600 ng of labeled RNA was fragmented and hybridized to the microarray according to Agilent's two-color microarray protocol. Two biological replicates were applied in this experiment.

### Microarray data analysis

Microarray slides were scanned using the Agilent DNA Microarray Scanner. Median foreground and background signals of the Cy3 and Cy5 channel were loaded into the R environment and processed using the LIMMA package [51]. Spots were down-weighted if 2 or more quality flags were set by the scanner software. Background signals were subtracted using the normexp method [52], Cy3 and Cy5 signals were Loess normalized and finally quantile normalisation was performed on all microarrays from one dataset. Signals from replicate probes for single genes were averaged. A linear model was fitted for each comparison of interest. The obtained p-values were adjusted for false discovery rate (fdr) using the method by Benjamini and Hochberg [53]. For further analysis, only those genes with a false-discovery-rate-adjusted p-value < 0.05 and an absolute log_2_ fold change > 1 under at least one condition were taken into account. Raw and processed microarray data have been deposited at the gene expression omnibus database under the accession number GSE47451.

### Validation of microarray data by qRT-PCR

Reverse transcription of the same RNA samples as those used for microarray analysis was performed with the QuantiTect Reverse Transcription Kit (Qiagen, Hilden, Germany), according to the manufacturer’s protocol. qPCR analysis was performed using the LightCycler 480 (Roche, Mannheim, Germany) with the QuantiTect SYBR Green PCR Kit (Qiagen, Hilden, Germany). Primer sequences are listed in Additional file 8: Table S4. Relative expression was calculated by the ∆∆C_t_ method and normalized to the *dapB* gene coding for dihydrodipicolinate reductase. *dapB* was selected as reference gene based on the microarray result, as it shows similar expression in all strains. The experiment was performed in duplicate.

### Identification of transcription factor binding sites (TFBS)

For the identification of binding sites for the transcription factor CtrA, promoter-regions ranging from 400 bp upstream to 50 bp downstream of the translation start were searched for matches with a position weight matrix (PWM) obtained from a comparative phylogenetic analysis of cell cycle regulation in *Alphaproteobacteria*[11]. A TFBS was considered as identified if its score reached at least 85% of the maximum weight of the PWM.

### Extraction and detection of AHLs

The production of AHLs was detected using the biosensor strain *P. putida* F117 pKR-C12 as previously described [24] with the following modifications. All *D. shibae* strains were grown in 100 ml of SWM with 5 mM succinate and 4% of adsorber resins (Amberlite XAD-16, Rohm & Haas) at 30°C and 160 rpm for 48 hours. Adsorber resins were harvested after 2 days by filtration and transferred to a separating funnel together with 50 mL distilled water, and then those resins were extracted with 25 ml of dichloromethane (DCM). The mixture was shaken vigorously for 1 min and the phases were allowed to separate. The DCM fraction was removed and another 25 ml of DCM was added. The whole extraction process was repeated three times. The combined DCM fractions were concentrated to 2 ml using a rotary evaporator (Heidolph VV2001, Schwabach, Germany) and stored at -20°C until the bioassay was conducted. For bioassays, 20 μl of the concentrated extract was applied to each well of a 96 well polypropylene micro-titer plate (PlateOne, Starlab, Hamburg, Germany). After air-drying, the micro-titer plate was overlaid with 100 μl of medium and 100 μl of the sensor strain and then incubated at 30°C with gentle agitation for 24 hours. Green fluorescence and optical density at 620 nm were determined in a Victor3 1420 Multilabel counter (PerkinElmer, Waltham, USA). The presence of AHLs in the strains was calculated by dividing the specific fluorescence (gfp_535_/OD_620_) of extracts by that of the negative control (DCM).

### GC/MS analysis

A volume of 1 μL of the extract was injected into an Agilent GC 7890A gas chromatograph connected to a 5975C mass-selective detector (Agilent) fitted with a HP-5 MS fused silica capillary column (30 m × 0.25 mm i.d., 0.22 μm film; Hewlett-Packard, Wilmington, USA). Conditions were as follows: inlet pressure: 67.5 kPa, He 24.2 ml min-1; injection volume: 1 μl; injector: 250°C; transfer line: 300°C; electron energy: 70 eV. The GC was programmed as follows: 50°C (5 min isothermic), increasing at 5°C min-1 to 320°C, and operated in splitless mode; carrier gas (He): 1.2 ml min-1. Retention indicies I were determined from a homologous series of n-alkanes (C8-C32).

### Distribution of CtrA-binding sites in QS gene promoters in *Alphaproteobacteria*

Finished genomes of *Alphaproteobacteria* with at least one gene with an autoinducer synthesis domain (pfam00765) and a *ctrA* homolog present were selected from IMG (http://img.jgi.doe.gov/). Promoter regions ranging from 400 bp up to 50 bp downstream of the start codon were downloaded for all genes with an autoinducer synthesis (pfam00765) or an autoinducer binding (pfam03475) domain (Additional file 9: Dataset S1). These regions were searched for putative *ctrA* binding sites the same way as described for the *D. shibae* genes.

### CtrA phylogeny in *Alphaproteobacteria*

The evolutionary history of CtrA protein sequences found in Additional file 10: Dataset S2 was inferred using the Neighbor-Joining method [54]. The optimal tree with the sum of branch length = 3.83257521 is shown. The tree is drawn to scale, with branch lengths in the same units as those of the evolutionary distances used to infer the phylogenetic tree. The evolutionary distances were computed using the Poisson correction method [55] and are in the units of the number of amino acid substitutions per site. The analysis involved 93 amino acid sequences. All positions containing gaps and missing data were eliminated. There were a total of 179 positions in the final dataset. Evolutionary analyses were conducted in MEGA5 [56].

## Electronic supplementary material

Additional file 1: Table S1: Expression data of *ctrA*, *cckA, chpT* and *luxI*_*1*_knockouts vs. wild-type in the exponential and the stationary phase. (XLSX 1 MB)

Additional file 2: Figure S1: Validation of microarray results by qRT-PCR. Differential expression of the genes *luxR*_*2*_ *, fliC, flgE* in the exponential growth phase and *rpoH*_*2*_in the stationary phase was validated using qRT-PCR. The log_2_ fold change of gene expression from microarray and qRT-PCR experiments for the mutant strains **(A)** *∆ctrA*, **(B)** *∆cckA* and **(C)** *∆chpT* versus wild-type strain are shown. Microarray results are indicated in blue and qRT-PCR results in red. The results represent the mean of two biological replicates. (TIFF 4 MB)

Additional file 3: Table S2: List of CtrA binding sites in *D. shibae*. (ODS 19 KB)

Additional file 4: Figure S2: Phenotypic characterization of stationary phase cells. **(A)** Phase contrast microscopy of *D. shibae* DFL12 wild-type and mutant strains in the stationary phase. Scale bar represents 20 μm. **(B)** Flow cytometric representation of morphological differences between mutant and wild-type strains in the stationary phase based on size (FSC, forward scatter) and granularity (SSC, side scatter). Numbers in plots (top right quadrant) indicate percent larger cells in this area. **(C)** Comparing the chromosome equivalent profiles of the corresponding strains in the exponential (grey area) and stationary phases (black line) by flow cytometry of SYBR Green I-stained cells (50,000 events counted). The *x* and *y* axes are in log_2_ scale and represent fluorescence intensity and cell density. **(D)** Boxplot showing the differences in cell size of indicating strains in the exponential and stationary phase. (TIFF 1 MB)

Additional file 5: Figure S3: Gene expression of plasmid pDshi01 in the phosphorelay mutants. **(A)** Genomic context and Heat map of the repABC replicon and the adjacent toxin/antidote plasmid stabilization system. Genes that are not involved in plasmid core functions are indicated with dotted lines. Color bar represents the fold changes in log_2_ scale. **(B)** Log_2_ fold changes of all genes located on the plasmid pDshi01 both in the exponential and stationary phase plotted against a theoretical normal distributed dataset demonstrates the bias towards down-regulation in the *∆ctrA* strain. *ctrA*: red; *cckA*: green; *chpT*: blue. **(C)** Gel electrophoresis testing the existence of pDshi01 in the three mutant strains using two primer combinations. Plasmid DNA from the wild-type strain was used as positive control and DNA of *∆pDshi01*strain as well as water were used as negative controls. (TIFF 233 KB)

Additional file 6: Figure S4: GC-MS analysis of extracts from *D. shibae*. **(A)** Total ion chromatogram of *D. shibae* wild-type. The traces of the ions m/z 102 and 143, characteristic for AHLs, are shown. Mass spectra of **(B)** C14en-HSL, **(C)** 3-oxo-C14-HSL, **(D)** C18en-HSL and **(E)** C18dien-HSL. (TIFF 467 KB)

Additional file 7: Table S3: Strains and plasmids used in this study. (DOCX 17 KB)

Additional file 8: Table S4: Primers used in this study. (DOCX 15 KB)

Additional file 9: Dataset S1: LuxIR homologs in *Alphaproteobacteria*. (TXT 632 KB)

Additional file 10: Dataset S2: CtrA homologs in *Alphaproteobacteria*. (TXT 34 KB)
