# Supplementary figures and images for: The CtrA phosphorelay integrates differentiation and communication in the marine alphaproteobacterium Dinoroseobacter shibae
Source: BMC Genomics. 2014 Feb 13;15(1):130. doi: 10.1186/1471-2164-15-130 (PMC4046655; doi:10.1186/1471-2164-15-130)

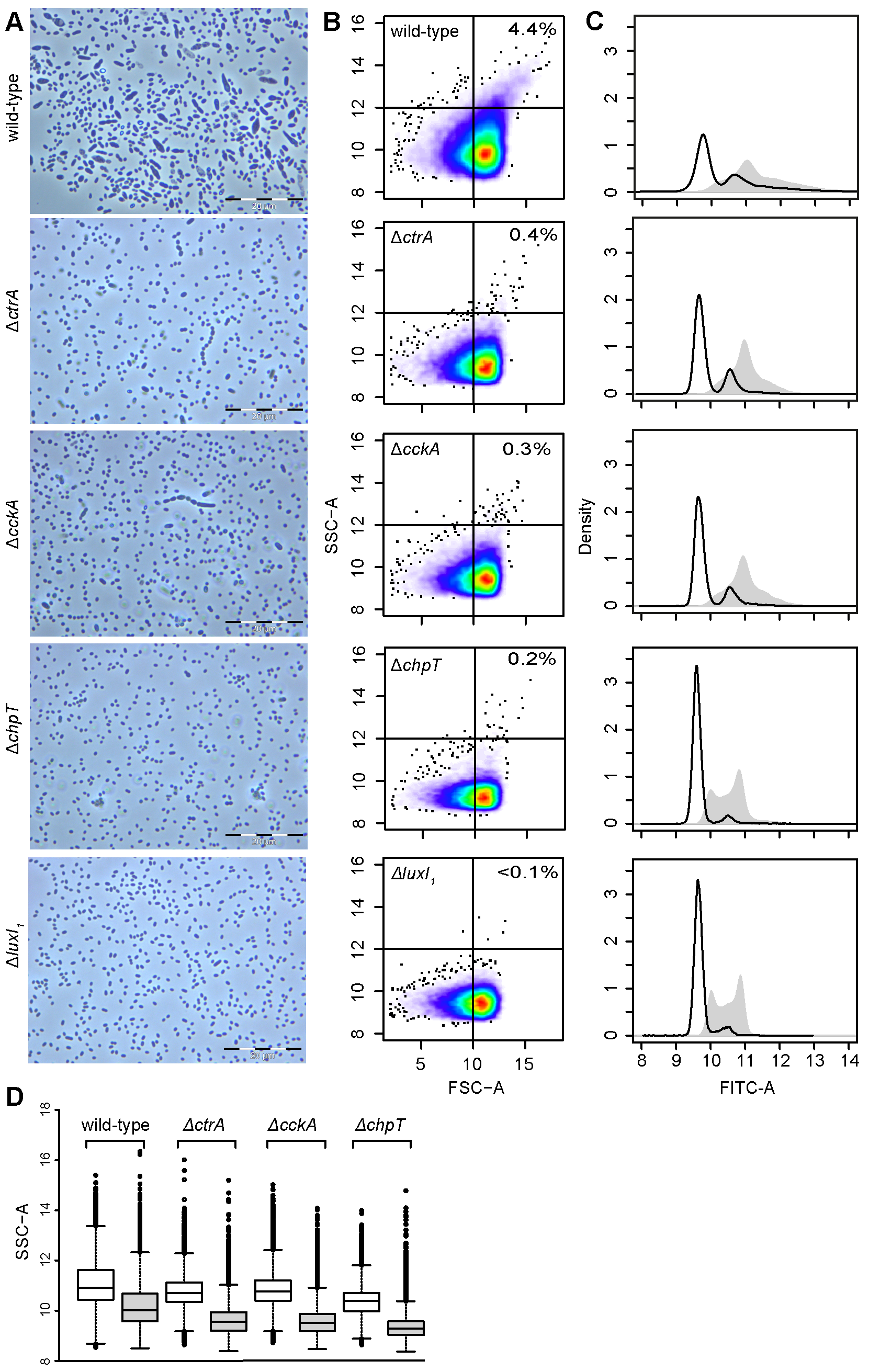

Supplement: Supplementary file 2 — Additional file 2: Figure S1: Validation of microarray results by qRT-PCR. Differential expression of the genes luxR 2 , fliC, flgE in the exponential growth phase and rpoH 2in the stationary phase was validated using qRT-PCR. The log2 fold change of gene expression from microarray and qRT-PCR experiments for the mutant strains (A) ∆ctrA, (B) ∆cckA and (C) ∆chpT versus wild-type strain are shown. Microarray results are indicated in blue and qRT-PCR results in red. The results represent the mean of two biological replicates. (TIFF 4 MB) [file 12864_2013_5784_MOESM2_ESM.tiff]

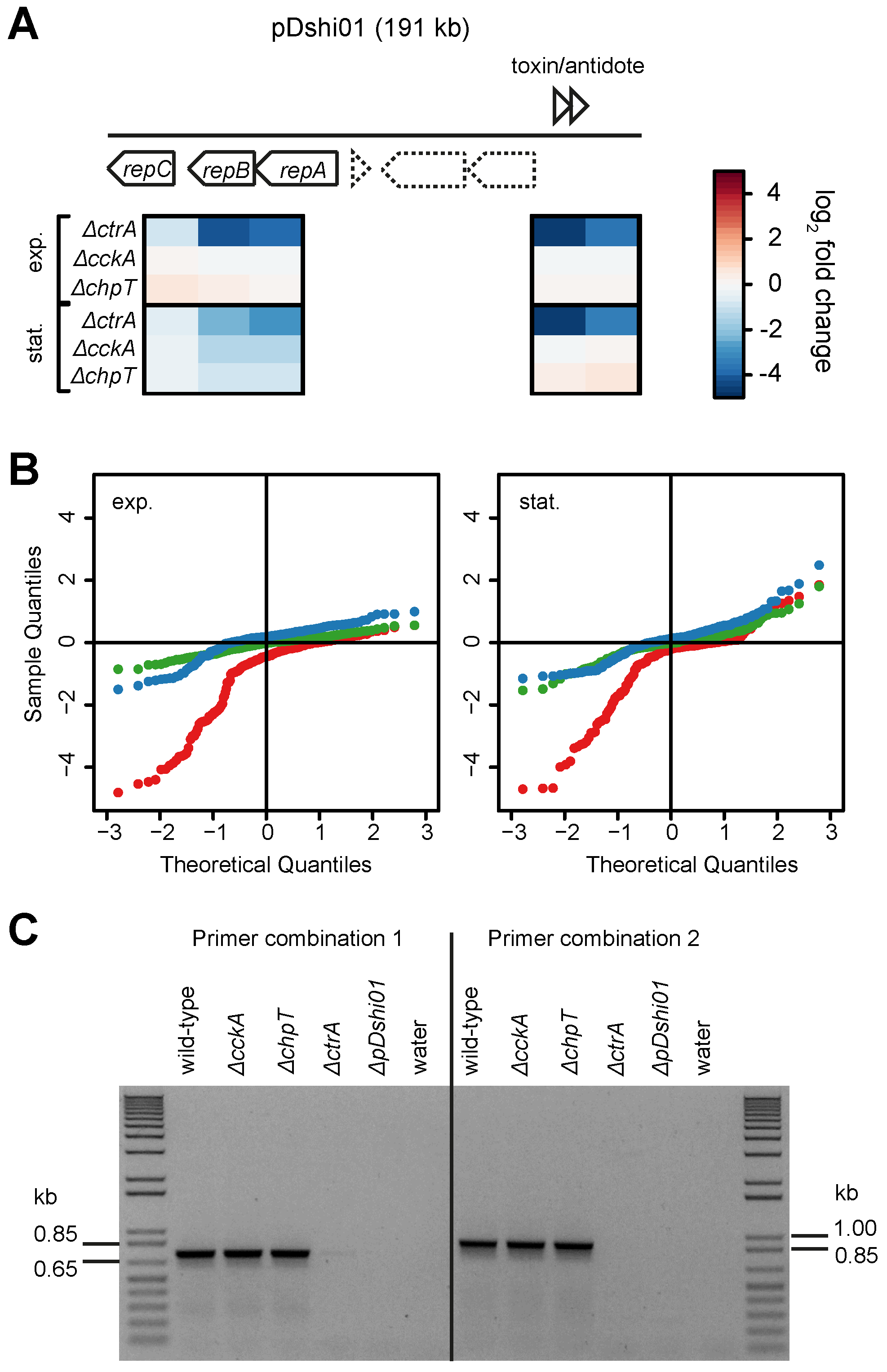

Supplement: Supplementary file 4 — Additional file 4: Figure S2: Phenotypic characterization of stationary phase cells. (A) Phase contrast microscopy of D. shibae DFL12 wild-type and mutant strains in the stationary phase. Scale bar represents 20 μm. (B) Flow cytometric representation of morphological differences between mutant and wild-type strains in the stationary phase based on size (FSC, forward scatter) and granularity (SSC, side scatter). Numbers in plots (top right quadrant) indicate percent larger cells in this area. (C) Comparing the chromosome equivalent profiles of the corresponding strains in the exponential (grey area) and stationary phases (black line) by flow cytometry of SYBR Green I-stained cells (50,000 events counted). The x and y axes are in log2 scale and represent fluorescence intensity and cell density. (D) Boxplot showing the differences in cell size of indicating strains in the exponential and stationary phase. (TIFF 1 MB) [file 12864_2013_5784_MOESM4_ESM.tiff]

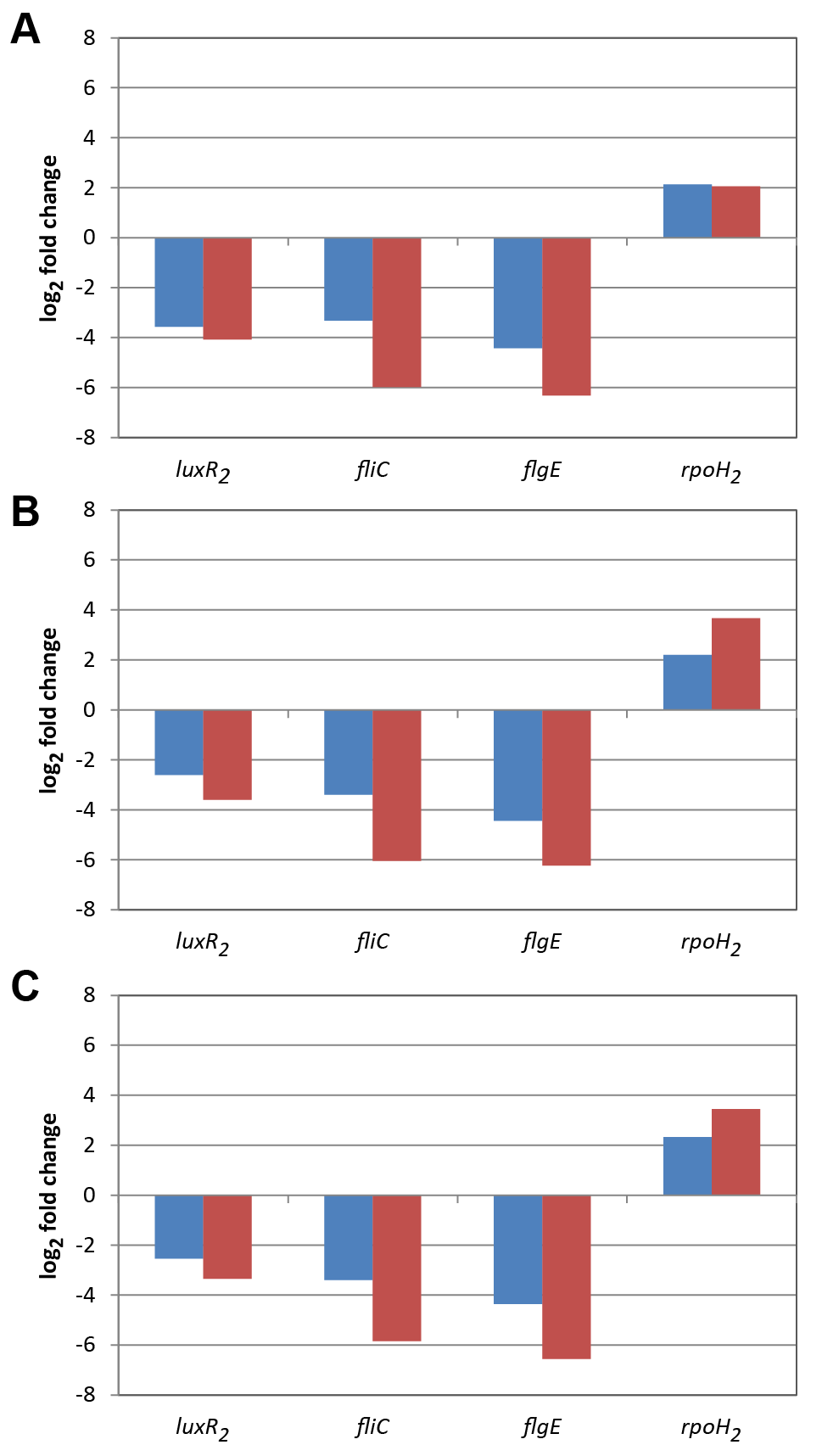

Supplement: Supplementary file 5 — Additional file 5: Figure S3: Gene expression of plasmid pDshi01 in the phosphorelay mutants. (A) Genomic context and Heat map of the repABC replicon and the adjacent toxin/antidote plasmid stabilization system. Genes that are not involved in plasmid core functions are indicated with dotted lines. Color bar represents the fold changes in log2 scale. (B) Log2 fold changes of all genes located on the plasmid pDshi01 both in the exponential and stationary phase plotted against a theoretical normal distributed dataset demonstrates the bias towards down-regulation in the ∆ctrA strain. ctrA: red; cckA: green; chpT: blue. (C) Gel electrophoresis testing the existence of pDshi01 in the three mutant strains using two primer combinations. Plasmid DNA from the wild-type strain was used as positive control and DNA of ∆pDshi01strain as well as water were used as negative controls. (TIFF 233 KB) [file 12864_2013_5784_MOESM5_ESM.tiff]

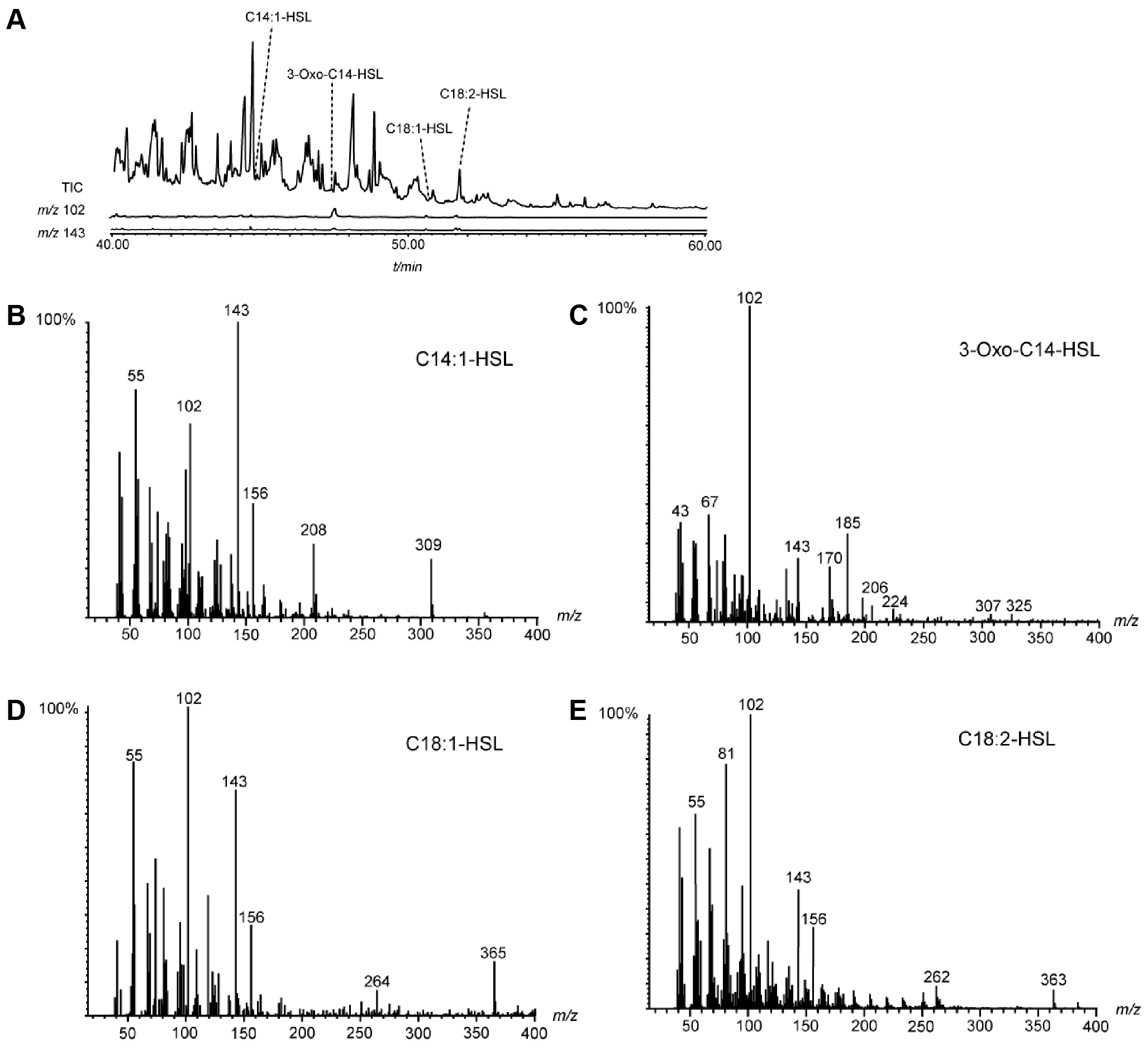

Supplement: Supplementary file 6 — Additional file 6: Figure S4: GC-MS analysis of extracts from D. shibae. (A) Total ion chromatogram of D. shibae wild-type. The traces of the ions m/z 102 and 143, characteristic for AHLs, are shown. Mass spectra of (B) C14en-HSL, (C) 3-oxo-C14-HSL, (D) C18en-HSL and (E) C18dien-HSL. (TIFF 467 KB) [file 12864_2013_5784_MOESM6_ESM.tiff]
