# Supplementary material for: The CtrA phosphorelay integrates differentiation and communication in the marine alphaproteobacterium Dinoroseobacter shibae
Source: BMC Genomics. 2014 Feb 13;15(1):130. doi: 10.1186/1471-2164-15-130 (PMC4046655; doi:10.1186/1471-2164-15-130)
Supplement: Supplementary file 7 — Additional file 7: Table S3: Strains and plasmids used in this study. (DOCX 17 KB) [file 12864_2013_5784_MOESM7_ESM.docx]

**Table S3. Strains and plasmids used in this study.**

| **Strain or Plasmid** | **Description** | **Source /Reference** |
| --- | --- | --- |
| ***D. shibae* strains** |  |  |
| DFL-12 | Wild-type | Biebl et al., 2005 |
| ∆*luxI_1_* | ∆*luxI_1_*:: Gm^r^ | Patzelt et al., 2013 |
| *∆ctrA* | *∆ctrA* :: Gm^r^ | This study |
| *∆ctrA*pHW1 | *∆ctrA* :: Gm^r^, Ka^r^ | This study |
| *∆cckA* | *∆cckA* :: Gm^r^ | This study |
| ∆*cckA*pHW2 | *∆cckA* :: Gm^r^, Ka^r^ | This study |
| *∆chpT* | *∆chpT* :: Gm^r^ | This study |
| ∆*chpT*pHW3 | *∆chpT* :: Gm^r^, Ka^r^ | This study |
| *E. coli* strains |  |  |
| DH5α | endA1 hsdR1 [r_K_-m_K_^+^] *glnV44 thi-1recA1 gyrA relA* ∆[*lacZYA-argF*] *U169 deoR* [ф80*dlac* ∆[*lacZ*]M15] | Herrero et al., 1990 |
|  |  |  |
| ST18 | S17 *λpir* ∆*hemA* | Thoma and Schobert, 2009 |
| *P. putida Strain* |  |  |
| F117 (pKR-C12) | AHL biosensor strain | Riedel et al., 2001 |
|  |  |  |
| **Plasmids** |  |  |
| pEX18Ap | Ap^r^; oriT^+^, sacB^+^, *lacZ, suicide vector* | Hoang et al., 1998 |
| pBBR1MCS-2 | Ka^r^; *lacZ* P*_lac_* P*_T7_ rep* | Kovach et al., 1995 |
| pBBR1MCS-5 | Gm^r^; *lacZ* P*_lac_* P*_T7_ rep* | Kovach et al., 1995 |
| pHW1 | Ka^r^; pBBR1MCS-2-*ctrA* | This study |
| pHW2 | Ka^r^; pBBR1MCS-2-*cckA* | This study |
| pHW3 | Ka^r^; pBBR1MCS-2-*chpT* | This study |

**References**

Biebl H, Allgaier M, Tindall BJ, Koblizek M, Lünsdorf H, Pukall R, Wagner-Döbler I (2005) *Dinoroseobacter shibae* gen. nov., sp. nov., a new aerobic phototrophic bacterium isolated from dinoflagellates. Int J Syst Evol Microbiol 55: 1089-1096.

Herrero M, de, L, V, Timmis, KN. (1990). Transposon Vectors Containing Non-Antibiotic Resistance Selection Markers for Cloning and Stable Chromosomal Insertion of Foreign Genes in Gram-Negative Bacteria. J Bacteriol 172(11):6557-67.

Hoang TT, Karkhoff-Schweizer RR, Kutchma AJ, Schweizer HP (1998) A broad-host-range Flp-FRT recombination system for site-specific excision of chromosomally-located DNA sequences: application for isolation of unmarked *Pseudomonas aeruginosa* mutants. Gene 212: 77-86.

Kovach ME, Elzer PH, Hill DS, Robertson GT, Farris MA, Roop RM, Peterson KM (1995) Four new derivatives of the broad-host-range cloning vector pBBR1MCS, carrying different antibiotic-resistance cassettes. Gene 166: 175-176.

Riedel K, Hentzer, M, Geisenberger, O, Huber, B, Steidle, A, Wu, H et al. (2001). N-Acylhomoserine-Lactone-Mediated Communication Between Pseudomonas Aeruginosa and Burkholderia Cepacia in Mixed Biofilms. Microbiology 147(Pt 12):3249-62.

Thoma S, Schobert M (2009) An improved Escherichia coli donor strain for diparental mating. FEMS Microbiol Lett 294: 127-132.
