# Supplementary material for: The CtrA phosphorelay integrates differentiation and communication in the marine alphaproteobacterium Dinoroseobacter shibae
Source: BMC Genomics. 2014 Feb 13;15(1):130. doi: 10.1186/1471-2164-15-130 (PMC4046655; doi:10.1186/1471-2164-15-130)
Supplement: Supplementary file 8 — Additional file 8: Table S4: Primers used in this study. (DOCX 15 KB) [file 12864_2013_5784_MOESM8_ESM.docx]

| **Table 4. Primers used in this study.** | |
| --- | --- |
| **Primer** | **Sequence 5' -> 3'** |
| **Primers for cloning** | |
| 800_ctrA_800-f | ATTCTCGTAGTCCAGCACGTAA |
| 800_ctrA_800-r | GGCGTGCGACATATAGACCT |
| down_ctrA-f | CGGTTGACCCCTCCACTC |
| up_ctrA-r | GGTCGTCTTCGATGATCAGG |
| ctrA_Gm-f | TCATCGAAGACGACC GACGCACACCGTGGAAA |
| ctrA_Gm-r | TGGAGGGGTCAACCG GCGGCGTTGTGACAATTT |
| Gm_resi_cass-f | GACGCACACCGTGGAAA |
| Gm_resi_cass-r | GCGGCGTTGTGACAATTT |
| 800_cckA_800-f | AAGCCCTTTATGAAGACGGAAT |
| 800_cckA_800-r | GATGAAGATCACCGTGCAGTT |
| up_cckA-r | CTCACTCTGTCCTCCGGTTC |
| dw_cckA-f | GTCAAAGACGCGAGCTAACC |
| cckA_Gm-f | GGAGGACAGAGTGAG GACGCACACCGTGGAAA |
| cckA_Gm-r | GCTCGCGTCTTTGAC GCGGCGTTGTGACAATTT |
| 800_chpT_800-f | ATCCCGTAGGCTTCGACATATT |
| 800_chpT_800-r | AGATGCTGACCATGACCGATAC |
| up_chpT-r | AGGCTCATCCATAGCAACCTC |
| dw_chpT-f | GGGAATGCCATCTCCGTA |
| chpT_Gm-f | GCTATGGATGAGCCT GACGCACACCGTGGAAA |
| chpT_Gm-r | GGAGATGGCATTCCC GCGGCGTTGTGACAATTT |
| ctrA-c-f | ATCTGCCGGATCGCAATA |
| ctrA-c-r | TTCGACATGCGAAGAACG |
| cckA-c-f | GACTGCTTGGTTCTTGCAG |
| chpT-c-f | ACCACCTTTCCCTCGTTCTC |
| chpT-c-r | AAATACCTGGTGGAGGGTGA |
| **Primers for qRT-PCR** | |
| dapB-f | GTGCATGTCATCGGCACTAC |
| dapB-r | CAGCTTCACAAGCAGGTTGA |
| luxR2-f | CTGGAAGATTTGCAGGAAGC |
| luxR2-r | CGATGCATCCAGACAGTGAT |
| rpoH2-f | TCCCAGAAGTCGCTGTTCTT |
| rpoH2-r | CATCTCGACGTCATGCAGAG |
| fliC-f | CAGCATTTTGACAAACAACAGC |
| fliC-r | ACCTTGGAGATCGACCAGATAG |
| flgE-f | GACCACGGAAACAGGTATGG |
| flgE-r | AGATTGACGGTGATCGGTTC |
| ctrA-f | TGACTACGATCTGATCCTGCTG |
| ctrA-r | GCCGAAACCCTTTATCTTGCTT |
| cckA-f | CGCAGGACAAGATCACGAAG |
| cckA-r | AAAATGAAGCCACCGGACTG |
| chpT-f | GTTTTGCCTATGACGCGACC |
| chpT-r | CCTCGATACGGAGATGGCAT |
